# Impact of Interventions on Medication Adherence in Patients With Coexisting Diabetes and Hypertension

**DOI:** 10.1111/hex.70010

**Published:** 2024-09-09

**Authors:** Pauline Tendai Maniki, Betty Bouad Chaar, Parisa Aslani

**Affiliations:** ^1^ Faculty of Medicine and Health, The University of Sydney School of Pharmacy The University of Sydney Sydney Australia

**Keywords:** disease management, multimorbidity, nonadherence, patient education, persistence

## Abstract

**Background:**

The coexistence of diabetes and hypertension is prevalent due to shared risk factors. Pharmacological treatment has been reported to be effective in managing both conditions. However, treatment effectiveness depends on the extent to which a patient adheres to their treatment. Poor adherence to long‐term treatment for chronic diseases is a growing global problem of significant magnitude. Several interventions have been developed to help improve medication adherence in patients with coexisting diabetes and hypertension. This review aimed to determine the characteristics of these interventions and their impact on medication adherence.

**Methods:**

A systematic review of the literature was conducted using the PRISMA guidelines and registered in the PROSPERO International Registry of Systematic Reviews. Studies were searched in the databases CINAHL, Embase and Medline to identify relevant articles published during 2012–2023. The search concepts included ‘medication adherence’, ‘hypertension’, ‘diabetes’ and ‘intervention’. Studies were included if they were in English and evaluated the impact of an intervention aimed at promoting adherence to medications for both diabetes and hypertension.

**Results:**

Seven studies met the inclusion criteria, with five demonstrating a statistically significant improvement in medication adherence. Of the five studies that improved medication adherence, four were multifaceted and one was a single‐component intervention. All successful interventions addressed at least two factors influencing non‐adherence. Patient education was the foundation of most of the successful interventions, supported by other strategies, such as follow‐ups and reminders.

**Conclusion:**

Multifaceted interventions that also included patient education had a positive impact on medication adherence in patients with coexisting diabetes and hypertension. Improving adherence in patients with coexisting diabetes and hypertension requires a multipronged approach that considers the range of factors impacting medication‐taking.

**Patient or Public Contribution:**

This systematic review provides comprehensive insights into the benefits of patient‐centred approaches in intervention development and strengthening. Such patient involvement ensures that medication adherence interventions are more relevant, acceptable and effective, ultimately leading to better health outcomes and more meaningful patient engagement in healthcare research.

## 1 Introduction

Diabetes and hypertension are common risk factors for cardiovascular disease (CVD), which is a leading cause of death and disability worldwide [1, 2, 3]. The coexistence of diabetes and hypertension is very common and has been attributed to metabolic pathophysiological pathways and shared risk factors such as obesity and endothelial dysfunction [2, 4]. The presence of either of the two conditions doubles the risk of developing the other [5, 6]. For instance, the incompetency of insulin in diabetes leads to the accumulation of glucose in the bloodstream, which can damage blood vessels and kidneys, resulting in high blood pressure [4, 5].

Pharmacological treatment decreases complications and premature death associated with diabetes and hypertension [7, 8]. However, treatment effectiveness is highly dependent on the patient's level of medication adherence [9]. Medication adherence is generally defined as ‘the process by which patients take their medications as prescribed' [10]. Due to suboptimal treatment adherence, the pharmacological effect of medications seen in randomized‐controlled trials can be less pronounced in real‐world settings [11, 12].

Poor adherence to medication is a worldwide public health concern, particularly for chronic conditions, with estimated general non‐adherence rates at approximately 50% [13]. Poor adherence is strongly associated with an increased economic and resource burden at both the health system and the patient level [6]. Various barriers to adherence have been identified and these include forgetfulness, poor communication between healthcare provider and patient, low health literacy and lack of conviction on the need for treatment [2, 14].

Patients with coexisting diabetes and hypertension face numerous challenges to their health, as these conditions affect different organ systems, which results in them needing two or more medications [2, 3, 6]. Being on multiple medications increases the risk of undesirable side effects, adverse reactions and increased medical expenditure [3, 8]. These risks also lead to poor adherence, especially in patients with limited knowledge of their condition [3, 6, 15]. Globally, adherence to medication therapy ranges between 36% and 93% in patients with coexisting diabetes and hypertension [8].

Good medication adherence is strongly associated with a high level of disease control [6, 11]. The level of disease control for hypertension and diabetes is reported to be low in patients with coexisting diabetes and hypertension [6]. For instance, Song et al. [16] reported a control rate of 40% and 41% for hypertension and diabetes, respectively, among patients aged 20 years and older with coexisting diabetes and hypertension at a hospital in China. The World Health Organization (WHO) identifies improving medication adherence as a more direct way to influence patient outcomes than addressing treatment improvements [17].

Addressing medication adherence for both conditions collectively is vital as the two conditions are closely linked such that each condition can potentially worsen the other [4, 5]. For instance, uncontrolled hypertension can exacerbate diabetes‐related complications such as kidney disease and neuropathy [4, 5]. On the other hand, poorly controlled diabetes increases vascular damage, making hypertension harder to control [4, 5]. Addressing medication adherence for both conditions collectively can ensure a more coordinated and effective approach to patient care that not only leads to better health outcomes but also cost‐effective treatment plans [18].

Medication non‐adherence is multifactorial and involves patients, healthcare providers, health systems, families and communities [8, 15]. Determinants of adherence are well established and several interventions have been developed to improve medication adherence in a range of diseases [19]. Several interventions have been designed to help patients manage their medication regimens more effectively, such as dispensing aids (e.g., pill boxes and webster packs) [20, 21, 22]. Digital innovations have also expanded the range of interventions through the development of mobile health applications and wearable devices that remind patients to take their medication [15]. Other interventions that have been developed target motivating and educating patients such as incentive payment, group sessions, patient education and follow‐up [15, 19].

These interventions have not been effective in all cases, and importantly, are not sustainable long term. For instance, incentives have been successful in improving medication adherence, but the effect was not sustainable after the incentives were withdrawn [23]. Similarly, many interventions require frequent communication and active monitoring, which may be impractical for most healthcare providers [15, 19].

Despite the increased incidence of coexisting diabetes and hypertension, most studies report on interventions to address medication adherence in diabetes and hypertension individually. This study will contribute to the body of knowledge on the impact of interventions on medication adherence in patients with coexisting diabetes and hypertension. To the best of our knowledge, no systematic review has focused on the impact of interventions on medication adherence in patients with coexisting diabetes and hypertension. Therefore, this systematic review aimed to

1. investigate the characteristics of interventions used to improve medication adherence in patients with coexisting diabetes and hypertension and
2. determine the impact of these interventions on improving medication adherence in patients with coexisting diabetes and hypertension.

## 2 Methods

A systematic review of the literature was conducted using the PRISMA guidelines and registered in the PROSPERO International Registry of Systematic Reviews (registration number—CRD42023423096).

### 2.1 Literature Search

We performed a literature search to identify research articles that assessed the impact of interventions on adherence to diabetes and hypertension medication in patients with coexisting diabetes and hypertension. Studies were searched in the following databases: CINAHL, Embase and Medline. Additional studies were identified through reference searching. Search terms included a combination of both controlled vocabulary and keywords such as ‘medication adherence’, ‘hypertension’, ‘diabetes’ and ‘intervention’. The complete search strategy is provided in Supporting Information S2: File A.

### 2.2 Eligibility Criteria

The search strategy was limited to studies published in English from June 2012 to December 2023 (inclusive). We limited the time of search to 2012–2023 because the Ascertaining Barriers to Compliance (ABC) taxonomy [10] for medication adherence that promotes consistency in taxonomy and methods used in studies addressing medication adherence was published in April 2012. Eligible studies fulfilled the following inclusion criteria: (1) intervention addressed medication adherence to therapeutic medication in both hypertension and diabetes, (2) medication adherence was one of the study outcomes and (3) published from June 2012 to December 2023 (inclusive). Studies that focused on medication adherence to prophylactic medication were excluded. Full texts were reviewed using the eligibility criteria mentioned above. Any disagreement between the two authors (P.T.M. and B.B.C.) was resolved by the third author (P.A.), and discussion until consensus was reached.

### 2.3 Data Extraction and Analysis

Covidence is a web‐based screening and data extraction tool for systematic reviews [24]. Data extraction was performed using a predesigned form generated through Covidence, which was edited to align with the review. We extracted information about (1) the author(s)/publication year, (2) study type, (3) country, (4) funding, (5) study setting, (6) type of intervention, (7) duration of intervention, (8) interventionist, (9) training of interventionist, (10) description of intervention, (11) non‐adherence determinants addressed by intervention, (12) adherence measuring tool and (13) effect of intervention on medication adherence. One author (P.T.M.) screened all studies based on the titles and abstracts and a second author (P.A.) screened 10% of the articles to check for any disagreements. Interventions were defined as all strategies designed to improve medication adherence as either a primary or secondary outcome.

**Figure 1:**
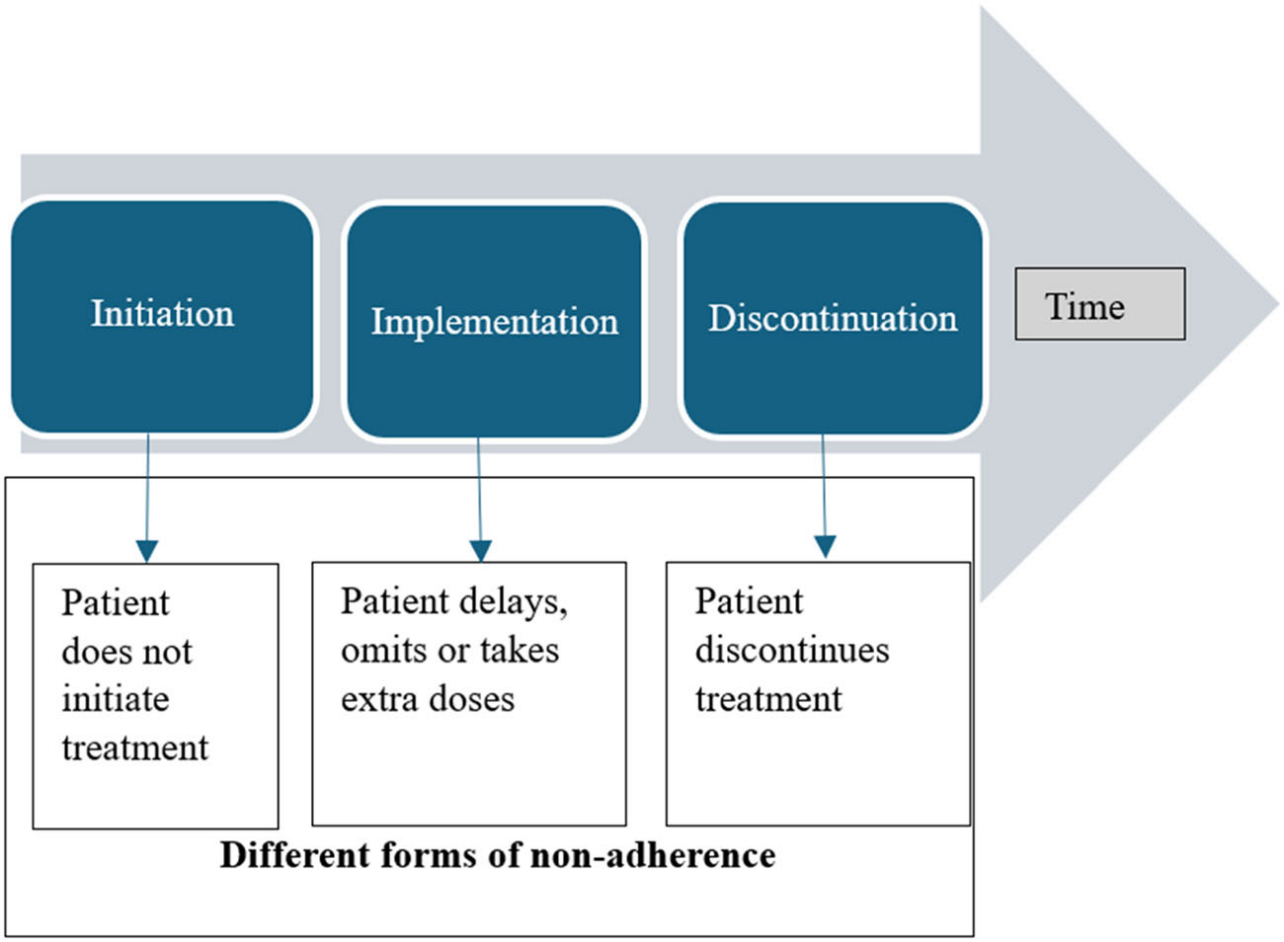
Medication adherence according to the ABC taxonomy: adapted from Vrijens et al. [10].

For each study included in the review, we identified the determinants of non‐adherence addressed by the intervention against the WHO classification: socioeconomic factors, healthcare team‐ and system‐related factors, therapy‐related factors, patient‐related factors and condition‐related factors [25]. In this review, medication adherence was defined as the degree to which individuals follow their prescribed medication regimen. We compared the definition used in the reviewed studies to the ABC taxonomy [10]. The ABC taxonomy defines medication adherence as ‘The process by which patients take their medication as prescribed’ and classifies the process into three phases, namely, initiation, implementation and discontinuation (Figure 1) [10]. Finally, we analysed additional outcomes linked to diabetes and hypertension management that were assessed in the studies.

### 2.4 Quality Assessment

The quality of studies was rated against the Covidence template for quality assessment (Risk of Bias), which is based on the Cochrane Risk of Bias version 1 tool [26]. Domains were scored as high (+) (good methodological safeguards to prevent bias), low (−) (poor methodological safeguards to prevent bias or unsure (?) (unclear methodological safeguards to prevent bias). Two independent authors (P.T.M. and B.B.C.) assessed all studies to determine the risk of bias. Any disagreement between the authors was resolved by the third author (P.A.).

## 3 Results

### 3.1 Study Selection

**Figure 2:**
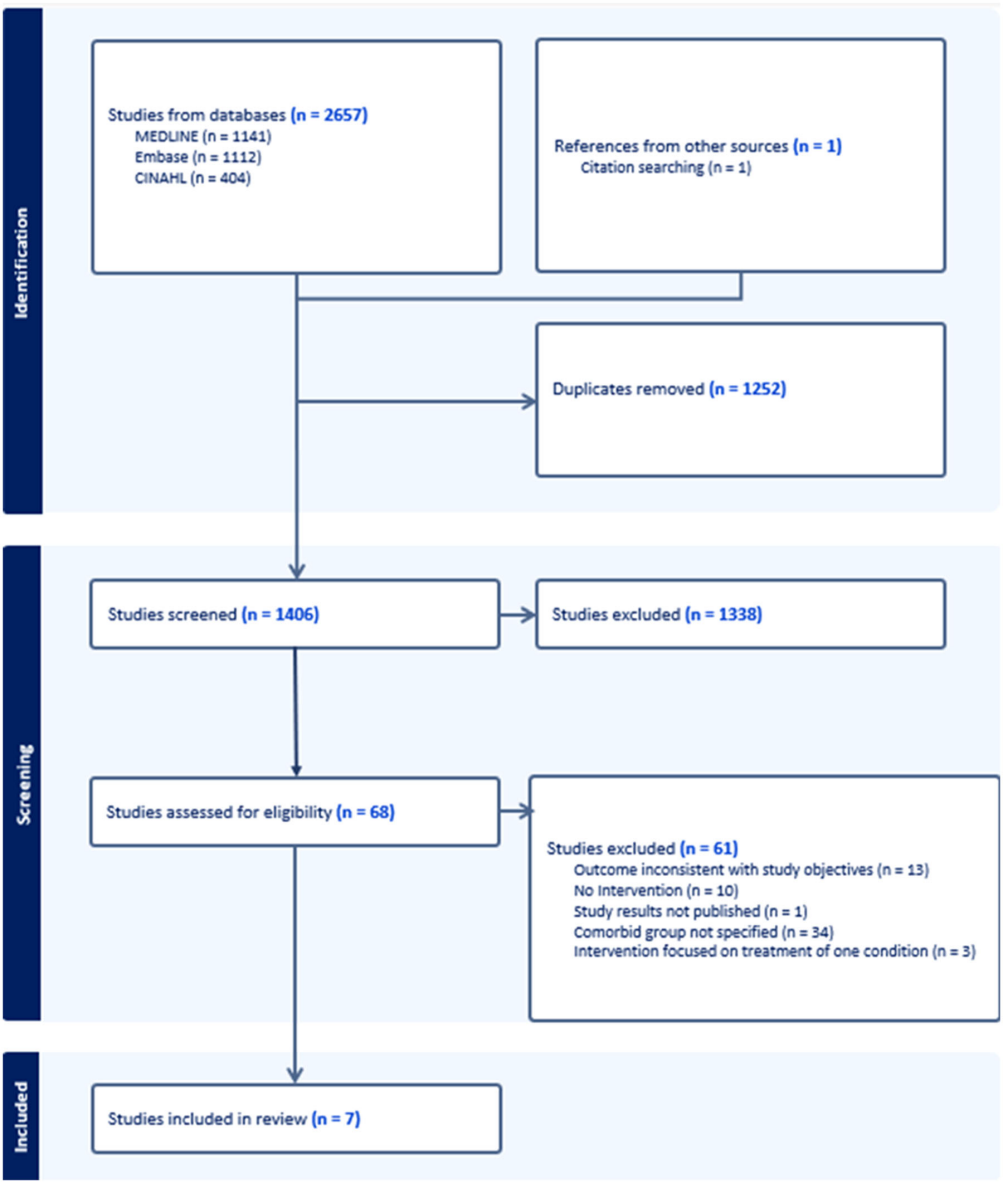
Flow diagram of the study selection process.

The literature search identified 2657 citations and one additional study was identified through reference searching, as illustrated in Figure 2. Of these, 67 appeared to meet the inclusion criteria and were retrieved in full text. Seven articles met the study inclusion criteria and were analysed.

### 3.2 Quality Appraisal

Four studies with a positive score on at least four domains were recorded as high‐quality studies, whereas three studies were classified as low quality as illustrated in Table 1. There was limited or no information on the blinding of people analysing the outcomes from intervention allocation in the studies reviewed. Therefore, the reviewed studies received ratings indicating either a ‘low’ or ‘unsure’ level of safeguarding against bias on blinding of outcome assessment.

**Table 1:** Quality appraisal of included studies.

	Sequence generation	Allocation concealment	Blinding of participants and personnel	Blinding of outcome assessment	Incomplete outcome data	Selective reporting	Other sources of bias
DeLeon et al. [12]	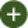	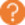	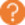	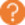	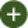	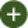	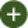
Soto et al. [8]	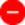	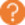	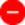	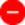	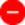	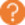	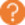
Moorhead et al. [15]	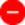	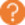	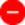	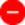	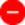	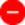	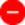
Kwayke et al. [3]	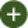	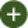	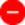	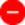	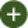	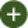	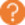
Contreras‐Vergara et al. [11]	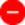	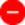	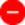	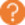	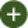	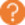	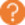
Malik et al. [2]	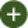	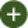	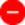	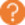	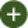	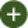	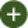
Wang et al. [6]	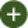	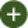	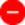	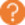	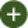	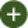	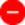

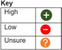

### 3.3 Characteristics of Included Studies

The studies included in the review were conducted in six countries, namely, United States [12, 15], Mexico [11], Ghana [3], Pakistan [2], Chile [8] and China [6]. Most (*n* = 4) [2, 3, 6, 11] were published recently (2021–2022), whereas the rest were published in 2015 [8, 12] and 2016 [15]. Three studies [2, 6, 11] were randomized‐controlled trials and the research design of one study [12] was not clear. Four studies [3, 8, 11, 15] recruited participants from outpatient departments, whereas others used community pharmacies [2], inpatient department [6] and prescription claims data [12]. Only Malik et al. [2] analysed medication adherence as a secondary outcome.

Inclusion criteria varied between studies and included age, diabetes or hypertension type, duration since diagnosis of both conditions and duration on medication. The minimum age for four studies [3, 6, 11, 15] was reported as 18 years and only two studies reported a maximum age limit of 60 [11] and 65 [6] years. Only Contreras‐Vergara et al. [11] limited the primary diagnosis based on hypertension type to systemic arterial hypertension and five [3, 6, 8, 11, 15] studies limited the diabetes type to type 2.

One study used duration since being diagnosed with both conditions [3] and another used duration on pharmacological treatment [8] as inclusion criteria. Disease control and clinical variables were used as inclusion criteria in three [2, 6, 15] of the studies, whereas the other four did not specify these [3, 8, 11, 12]. For example, Moorhead et al. [15] and Wang et al. [6] recruited participants with uncontrolled hypertension and diabetes, whereas Malik et al. [2] recruited participants who had HbA1c ≥ 7% and blood pressure > 140/90 at diagnosis. The smallest study population had *n* = 50 [8], whereas the largest assessed 3602 [12] prescription records. Table 2 presents the characteristics of the included studies.

**Table 2:** Characteristics of included studies.

Study	Study design	Sample size of participants who completed the study (Control group, Intervention group)	Age criterion	Condition specified	Outcomes analysed P—Primary S—Secondary
DeLeon et al. [12]	N/S	3602 (C = 2855, I = 747)	N/S	All types of diabetes and hypertension	P—Medication adherence
Soto et al. [8]	Prospective study	50	> 18 years	Type 2 diabetes and hypertension	P—Medication adherence, disease knowledge, BP and HbA1c S—BMI
Moorhead et al. [15]	Post hoc analysis	131 (57—efficacy assessment, 74—safety assessment)	≥ 18 years	Type 2 diabetes and hypertension	P—Medication adherence and risk of overdosing
Kwayke et al. [3]	Intervention study	328 (C = 187, I = 141)	≥ 18 years	Type 2 diabetes and hypertension	P—Medication adherence, BP, level of knowledge FBG, BMI and weight
Contreras‐Vergara et al. [11]	Randomized clinical trial	89 (C = 43, I = 46)	18–60 years	Type 2 diabetes and systemic arterial hypertension	P—Medication adherence S—FBG, HbA1c, blood pressure, cholesterol and triglyceride levels.
Malik et al. [2]	Randomized, controlled, single‐blind, pre–post‐intervention	80 (C = 40, I = 40)	≥ 20 years	Type 1 or 2 diabetes and hypertension	P—FBG and BP S—Medication adherence and patient knowledge.
Wang et al. [6]	Randomized‐controlled trial	80 (C = 40, I = 40)	18–65 years	Type 2 diabetes and hypertension	P—Medication adherence S—FBG, 2hPG, HbA1c, rate of reaching target BP and risk factor profile.

Abbreviations: 2hPG, 2‐h postprandial glucose; BP, blood pressure; C, control group; FBG, fasting blood glucose; HbA1c, glycated haemoglobin; I, intervention group; N/S, not specified.

### 3.4 Medication Adherence

Generally, all studies regarded medication adherence as the extent to which a patient follows a therapeutic regimen. None of the studies in this review used the medication adherence taxonomy or referred to the three adherence phases [10]. Even though all studies used the word ‘adherence’ to describe medication‐taking behaviour, some (*n* = 4) [2, 6, 11, 15] also used terms such as ‘compliance’ and ‘noncompliance’.

To measure medication adherence, most studies (*n* = 6) [2, 3, 6, 8, 11, 12] used indirect methods of measuring medication adherence, with two [8, 12] using objective methods such as pill counts and four [2, 3, 6, 11] using subjective methods such as patient questionnaires (full details about the methods of measuring medication adherence are provided in Appendix S1). The two studies [8, 12] that used objective methods did not report a statistically significant improvement in medication adherence. Five studies [3, 6, 8, 11, 15] combined medication adherence for both conditions, whereas De Leon et al. [12] and Malik et al. [2] evaluated adherence to hypertension and diabetes medications separately. Studies [2, 3, 6, 11, 15] that reported a statistically significant improvement in medication adherence were regarded as successful in this review.

### 3.5 Intervention Characteristics

Six studies [2, 3, 6, 8, 11, 12] specified the interventionists—pharmacists were the interventionists in five studies [2, 3, 6, 8, 11] whereas physicians were the interventionists in one study [12]. The provision of training to interventionists was only specified in three of the studies [2, 11, 12], whereas Wang et al. [6] recruited one pharmacist with 10 years of professional experience to evaluate medication adherence. One study [12] evaluated the impact of an intervention on patients' medication adherence, where the intervention was delivered to the interventionist (physicians). All other studies delivered an intervention directly to the patient participants and evaluated the impact of the intervention on patient outcomes. Specifically, Malik et al. [2] focused on both training interventionists and delivering the intervention to the patient participants, and Contreras‐Vergara et al. [11] used a trained pharmacist to deliver the intervention to the intervention group and physicians to deliver regular education to the control group participants.

Almost half of the interventions lasted for 6 months (*n* = 3) [2, 3, 11] and the least intervention period was 4 weeks [15]. Most interventions (*n* = 5) [2, 3, 6, 8, 11] assessed were multifaceted, with the remaining using single‐component interventions (one educational and one behavioural). The interventions reviewed used a diverse range of strategies such as the provision of educational materials [2, 6, 11], decision‐making support to healthcare workers [2, 12], provision of patient aids [2, 6, 15], inclusion of family support [8] and use of telephone follow‐ups [3, 6] or social media follow‐ups [6].

The most addressed WHO dimensions [25] of non‐adherence were patient‐related (*n* = 6) [2, 3, 6, 8, 11, 15], healthcare team‐ and system‐related (*n* = 6) [2, 3, 6, 11, 15] and therapy‐related (*n* = 5) [2, 3, 6, 8, 11] factors. None of the studies covered all five dimensions of non‐adherence, in particular, the economic dimension of non‐adherence. Five studies [2, 3, 6, 11, 15] reported a statistically significant positive impact of their intervention on medication adherence in participants with co‐existing hypertension and diabetes. Table 3 summarizes the intervention characteristics of the included studies.

**Table 3:** Intervention characteristics and success.

Study Country WHO dimensions used	Adherence measurement details Method (M) Tool (T) Calculation (C)	Intervention description		Duration and frequency of intervention	Changes reported		Was medication adherence improved successfully?
		Intervention group	Control group		Baseline	Endpoint	
De Leon et al. [12] USA Healthcare system/team‐related	M: Prescription claims T: Medication possession ratio C: Sum of the days of prescription supply dispensed between the first and last pharmacy fill divided by the number of days between the prescriptions Adherent – MPR ≥ 80%	Physicians were supported in improving population health through e‐prescribing, provider reminders and clinical decision support. Physicians were trained to enhance their knowledge of health information technology and onsite consulting.	Physicians were not part of the Health Information and data exchange programme.	Physicians received training on a quarterly basis. Prescription claims data for 3 years were analysed in this study.	*Percentage of adherent members* *Diabetes‐specific:* I: 36.04% C: 37.37% *Hypertension‐specific:* I: 40.57% C: 44.44%	*Percentage of adherent members* *Diabetes‐specific:* I: 37.10% C: 37.87% *Hypertension‐specific:* I: 41.94% C: 47.76% (Both groups showed improvement but non‐statistically significant adherence, with the control group performing better on hypertension medication and the intervention group on diabetes medication)	No
Soto et al. [8] Chile Patient‐related, Therapy‐related, Socioeconomic‐related and Condition‐related	M: Pill Count C: (no. of pills dispensed—no. of pills returned)/number of pills prescribed × 100. Good adherence—Pill count between 80% and 110%.	Face‐to‐face interviews to educate patients on their disease and treatment. Pharmacotherapeutic plan tailored to patient needs. Calendar to prevent forgetfulness. Family support.	N/A	3 (30–45 min) consultations for 4‐6 months.	*Medication* a *adherence by %* Adherent participants: 15/50 (30%) Non‐adherent participants: 35/50 (70%)	*Medication* a *adherence by %* *Adherent participants: 23/50 (46%)* *Non‐adherent participants: 27/50 (54%)* (*Adherence increased from 30% to 46%, but this change was not statistically significant*)	No
Moorhead et al. [15] USA Patient‐related and Healthcare system/team‐related	M: Mean daily adherence—Binary variable that indicates if patient took medication before or after seeing dose reminder. T: Electronic measure C: mean daily adherence = number of pills detected by the wearable sensor patch divided by number of pills expected for each medicine. Daily on‐time adherence = number of pills for each medicine detected by wearable sensor patch within 2 h of dosing time divided by expected number of pills for each medicine.	Participants received sensor‐enabled reminders (medicines and dose) if within Bluetooth range, within 30 min of a scheduled dose	N/A	Either 4 or 12 weeks	Not reported	*Overall Adherence*:^a^ ≤ 60% = 2 participants > 60% to 70% = 2 participants > 70% to 80% = 8 participants > 80% to 100% = 45 participants (79% of the participants had consistently good adherence ≥ 80%)	Yes
Kwayke et al. [3] Ghana Patient‐related, Condition‐related, Therapy‐related and Socioeconomic‐related.	M: Self‐reported T: Medication adherence reasoning scale (MARS‐10). C: Score of 6 ≤ adherent and < 6 non‐adherent	Face‐to‐face interviews educating patients on disease, medication and lifestyle modification. Individualized supportive care tailored to patient needs. Continuous telephone follow‐up calls (Frequency unspecified).	Usual dispensing services and care. Follow‐up calls (Frequency Unspecified).	3 (10–20 min) at 0, 3 and 6 months	*Adherent members* a Baseline: I = 20.70% C = 24.26% Month 3: I = 30.47% C = 19.23%	*Adherent members*:^a^ I = 39.64% C = 22.19% [Difference in adherence between the two groups was significant at 3 and 6 months (endpoint)]	Yes
Contreras‐Vergara et al. [11] Mexico Patient‐related, Therapy‐related, Condition‐related and Healthcare system/team related	M: Self‐reported T: The 8‐item Morisky Medication Adherence Scale (MMAS‐8) C: High level of adherence score = 8, medium = (6–7), and low < 6.	Face‐to‐face consultations educating patients on their condition and treatment. Patients received wallet cards with updated lists of their medications. Recommendations for follow‐up care.	Regular standard education from physicians.	3 (20–25 min) at 0, 3 and 6 months	*Medication adherence*:^a^ *Mean ± SD* I = 4.5 ± 2.1 C = 4.9 ± 1.9 (Difference in adherence between the two groups was not significant at baseline and 3 months) 3 months mean adherence + SD not specified	*Medication adherence*:^a^ *Mean ± SD* I = 7.04 ± 1.4 C = 5.1 ± 1.4 (Difference in adherence between the two groups was significant at 6 months)	Yes
Malik et al. [2] Pakistan Patient‐related, Condition‐related, Therapy‐related and Healthcare system/team‐related	M: Self‐reported T: The Brief Medication questionnaire (BMQ) C: Sum of ranks—total ranking of responses. Regimen screen: Low sum of ranks—better medication adherence. Belief screen: High sum of ranks—more positive perception of medication. Recall screen: High sum of ranks—high ability to recall important details about medication regimen. Access screen: Low sum of ranks—improved access to medication.	Pharmacists were trained on disease management and given training aids. Patients received face‐to‐face counselling tailored to their needs and were educated on their condition and medication. Patients were given patient kits with disease brochures, BP and glucose monitoring cards and diet charts.	Pharmacists not trained Patients received usual pharmacy services	≥ 20‐min sessions every 15 days for 6 months	*Sum of ranks in the intervention group* *Diabetes* *Regimen screen*: 1921.0 Belief screen: 858.0 *Recall screen*: 820.0 *Access screen*: 1620.0 *Hypertension* *Regimen screen*: 1951.0 *Belief screen*: 1734.5 *Recall screen*: 1802.0 *Access screen*: 1665.0	*Sum of ranks in the intervention group* *Diabetes* *Regimen screen*: 1319.0 Belief screen: 2382.0 *Recall screen*: 2420.0 *Access screen*: 1620.0 *Hypertension* *Regimen screen*: 1288.5 *Belief screen*: 1505.5 *Recall screen*: 1438.0 *Access screen*: 1575.0 (The change was insignificant for recall screen on diabetes medication and belief and access screen for hypertension)	Yes
Wang et al. [6] China Patient‐related, Therapy‐related, and Healthcare system/team related	M: Self‐reported T: The Morisky–Green test (MGT). C: Good Adherence score = 4	Pharmaceutical care, personalized education form and materials on treatment and medication guidance provided after discharge. Consultation with clinical pharmacists every 2 weeks after physician visit. Medication time indicator charts. WeChat account where participants could ask pharmacists questions. Follow‐up by telephone every two weeks.	Routine clinical care after discharge Personalized education form on treatment at discharge Routine care. Follow‐up by telephone once a month.	Fortnight consultations, Daily access to Clinical pharmacist via WeChat and Follow‐up every 2 weeks for 3 months	Medication^a^ adherence rate (primary endpoint) I: 39 (97.5%) C: 38 (95.0%)	Medication^a^ adherence rate (primary endpoint) I: 36 (90.0%) C: 21 (52.5%)	Yes

a Medication adherence combined for diabetes and hypertension.

### 3.6 Characteristics of Interventions Reporting Improved Medication Adherence

Four [2, 3, 6, 11] out of the five [2, 3, 6, 11, 15] studies that showed a statistically significant improvement in medication adherence used multifaceted interventions. Pharmacists were the interventionists in these four studies. Training of interventionist [2, 11] or use of experienced interventionist [6] was reported in 60% of the successful studies. The factors related to non‐adherence that were targeted by the interventions showed a similar pattern. All successful interventions addressed at least two WHO dimensions of factors influencing non‐adherence, with the common ones being patient‐related [2, 3, 6, 11, 15], healthcare team/system‐related [2, 3, 6, 11, 15] and therapy‐related factors [2, 3, 6, 11].

Patient education was the cornerstone in 80% [2, 3, 6, 11] of the successful interventions. All successful interventions [2, 3, 6, 11, 15] focused mainly on delivering the intervention to the patient, with a particular emphasis on patient empowerment.

The common issue addressed under the healthcare team/system‐related dimension was improved communication between healthcare practitioners and patients and the training of interventionists. Interventions with a prolonged or consistent interaction between healthcare professionals and patients reported a statistically significant improvement in medication adherence. For example, Malik et al. [2] reported that the participants met with the pharmacists every 15 days for 6 months, whereas Wang et al. [6] reported a meet‐up every week, daily WeChat (Mobile messaging application) account access and continuous follow‐ups every 2 weeks.

### 3.7 Additional Outcomes Assessed

Six [2, 3, 6, 8, 11, 15] of the studies assessed additional outcomes and these included blood pressure, fasting plasma glucose (FPG), glycated haemoglobin (HbA1c), systolic blood pressure (SBP), diastolic blood pressure (DBP) and patient knowledge. All multifaceted [2, 3, 6, 8, 11] interventions assessed more than three additional outcomes linked to diabetes and hypertension management such as blood pressure and reported a significant improvement in at least three outcomes. This was not the same for the two single‐component interventions, as only Moorhead et al. [15] assessed risk of overdosing as the only additional outcome. Table 4 summarizes the additional outcomes linked to hypertension and diabetes management assessed in the included studies.

**Table 4:** Additional outcomes assessed linked to hypertension and diabetes.

Study	Changes reported			
	Outcome assessed	Baseline	Endline	*p* value
De Leon et al. [12]	N/A	N/A	N/A	
Soto et al. [8] {mean ± SD}	Disease knowledge (high‐answered ≥ 2 questions out of 3 correctly)	High: 5	High: 33	**< 0.0001**
		Low: 45	Low: 17	
	SBP (mmHg)	151.0 ± 19.0	135.0 ± 16.0	**< 0.0001**
	DBP (mmHg)	82.0 ± 12.0	78.0 ± 10.0	0.06
	HbA1c (%)	9.4 ± 2.3	8.8 ± 2.4	**< 0.0001**
	BMI (kg/m^2^)	34.6 ± 7.5	34.6 ± 7.7	0.89
Moorhead et al. [15]	Risk of overdosing	No cases of overdose related to DH medication dose reminders occurred		
Kwayke et al. [3] {Median value (and interquartile range)}	Medication knowledge (a score above 80% was classified as adequate).	I: 20.4%	I: 44.0%	**< 0.0001**
		C: 24.3%	C: 23.9%	
	SBP (mmHg)	I: 130.0 (120–150)	I: 120.0 (120–130)	**< 0.0001**
		C: 140.0 (120.0–150.0)	C; 130.0 (120.0–14.0)	
	DBP (mmHg)	I: 80.0 (80.0–90.0)	I: 80.0 (80.0–80.0)	**< 0.0001**
		C: 80.0 (80.0–90.0)	C: 80.0 (80.0–90.0)	
	FBG (mmol/L)	I: 8.1 (6.5–10.1)	I: 6.2 (5.5–7.0)	**< 0.0001**
		C: 7.5 (6.1–9.8)	C: 8.8 (7.3–10.7)	
	BMI (kg/m^2^)	I: 27.85(24.2–32.0)	I: 27.3 (23.9–31.2)	**0.005**
		C: 29.4(24.8–33.8)	C: 29.6 (24.6–33.9)	
	Weight (kg)	I: 75.0(64.3–86.0)	I: 73.0 (63.0–84.0)	**0.006**
		C: 77.0(70.0–89.0)	C: 77.5 (68.4–91.0)	
Contreras‐Vergara et al. [11) {mean ± SD}	SBP (mmHg)	I: 139.9 ± 10.14	I: 130.15 ± 11.8	I: 0.782
		C: 140.95 ± 13.7	C: 141.14 ± 13.1	C: **< 0.0001**
	DBP (mmHg)	I: 92.83 ± 6.01	I: 87 ± 3.83	I: **< 0.0001**
		C: 92.26 ± 6.12	C: 92.91 ± 6.25	C: 0.425
	FBG (mg/dL)	I: 148.5 ± 28.7	I: 139.2 ± 15.6	I: < **0.0001**
		C: 157.3 ± 47.6	C: 157.2 ± 38.2	C: 0.194
	HbA1c % (mmol/mol)	I: 9.07 (73) ± 1.76	I: 7.6 (60) ± 0.96	I: < **0.0001**
		C: 8.86 (73) ± 1.80	C: 8.80 (73) ± 1.34	C: 0.0436
Malik et al. [2] {mean ± SD}	Patient diabetes knowledge—Higher mean scores show better knowledge	I: 16.02 (± 2.93)	I: 19.97 (± 2.66)	0.003
		C: 13.95 (± 3.11)	C: 12.72 (± 3.47)	
	Patient hypertension knowledge—Higher mean scores show better knowledge	I: 15.60 (± 3.33)	I: 18.35 (± 2.31)	0.002
		C: 14.67 (± 2.48)	C: 14.32 (± 2.42)	
	FBG (mmol/L)	I: 11.92 (± 2.15)	I: 8.25 (± 1.48)	**< 0.0001**
		C: 11.60 (± 1.50)	C: 11.54 (± 1.56)	
	SBP (mmHg)	I: 145.85 (± 10.88)	I: 130.10 (± 6.89)	**< 0.0001**
		C: 142.15 (± 9.36)	C: 145.48 (± 6.69)	
	DBP (mmHg)	I: 95.08 (± 10.96)	I: 88.83 (± 5.38)	**< 0.0001**
		C: 95.00 (± 8.85)	C: 97.00 (± 6.90)	
Wang et al. [6] {Median (Interquartile range)}	Blood pressure achieving target rate, *n* (%)	I: 40 (100.0)	I: 37 (92.5)	**0.001**
		C: 40 (100.0)	C: 25 (62.5)	
	Blood glucose achieving target rate, *n* (%)	I: 32 (80.0)	I: 22 (55.0)	**0.032**
		C: 37 (92.5)	C: 12 (30.0)	
	FPG (mmol/L)	I: 6.40 (6.00, 7.00)	I: 6.50 (6.00, 7.18)	**0.004**
		C: 6.30 (6.00, 6.64)	C: 7.00 (6.83, 7.78)	
	HbA1c (%)	I: 7.10 (6.43, 7.80)	I: 6.45 (6.30, 7.00)	**0.007**
		C: 7.80 (6.63, 8.00)	C: 6.95 (6.50, 7.38)	
	2hPG (mmol/L)	I: 8.50 (8.05, 9.00)	I: 8.45 (7.45, 9.28)	**0.007**
		C: 8.85 (8.20, 9.20)	C: 9.35 (8.23, 10.15)	
	Adverse events reported	N/A	I: 0	0.055
			C: 5	

*Note:* Bold *p* value denotes statistically significant.

Patient knowledge assessment varied and the three studies that assessed this outcome looked at patient knowledge of medication [3], disease management [2] and the disease [8]. The interventions were successful in improving patient knowledge in all three studies. Of the five studies [2, 3, 6, 8, 11] that assessed blood pressure, four studies [2, 3, 8, 11] distinguished between SBP and DBP, and SBP decreased significantly in three studies [2, 3, 8], whereas DBP decreased in three studies [2, 3, 11]. Blood glucose was assessed using FPG [2, 3, 6, 11], HbA1c [2, 3, 6, 11] and 2‐h plasma glucose (2hPG) [6] and all studies reported a significant decrease post‐intervention. Finally, Contreras‐Vergara et al. [11] also assessed the relationship between disease control and medication adherence. However, a significant relationship was reported between medication adherence and blood pressure, but not blood glucose [11].

## 4 Discussion

This review identified a small number of studies that focused on medication adherence interventions in patients with co‐existing diabetes and hypertension. However, a majority of the interventions reported in these studies demonstrated a significant positive impact on medication adherence. Most of the successful interventions used a multifaceted strategy that targeted both educational and behavioural aspects of adherence. This is in line with previous literature and suggests that multifaceted interventions are more effective than single‐component interventions, as they appear to target both intentional and non‐intentional medication adherence [27, 28], and more than one factor impacting medication‐taking. Similarly, Sapkota et al. [28] found that multifaceted interventions were more successful in improving medication adherence than single strategies in patients with diabetes. In contrast, a study that assessed the impact of multifaceted interventions to support medication adherence after stroke did not report a positive impact [29]. However, this could be attributed to the high initial adherence in the study, leaving little room for further improvement.

We found that all successful interventions addressed patient‐related and healthcare professional/team determinants of non‐adherence. These findings underscore the importance of a patient‐centred approach to fostering medication adherence and maintaining a therapeutic alliance to support medication‐taking. A strong therapeutic alliance between the patient and the healthcare professional has been associated with improved medication adherence, as it leads to a more personalized approach to healthcare. The strength of a therapeutic alliance is reliant on both the patient and the healthcare professional [30, 31], as well as on effective communication [30]

Continuous communication between the patient and the health service provider was a consistent component of most of the successful interventions identified in this review. Paterick et al. [32] highlighted the importance of a continuous connection between health professionals and patients in the effective management of chronic diseases. Similarly, Williams et al. [33] emphasized that the flow of information between patients and health professionals should be consistent with the progression of the chronic disease. These findings suggest that continuous communication must be utilized in medication adherence interventions, as it could lead to more favourable outcomes and reinforce the sustainability of the intervention.

There was strong evidence for a positive effect of interventions using patient education as a strategy for improving patient adherence in this review. The cornerstone for medication adherence is patient education. Patient education enhances self‐management, which has long been recognized as an effective technique for improving medication adherence [34]. Various studies have associated enhanced patient education with improved medication adherence [30, 31, 35].

Moreover, all successful interventions focused mainly on empowering the patient, with a few also focusing partly on empowering the healthcare professional. Only De Leon et al. focused mainly on empowering the healthcare care providers to provide better care but did not report a significant change in medication adherence. Research evidence suggests that effective interventions should adopt a patient‐centred approach as a foundation for developing new, and enhancing existing, adherence interventions [32, 36]. A greater improvement in medication adherence can be expected from empowering patients to take on an active role in their healthcare management. However, this approach should be complemented by empowering healthcare providers to support and guide patients effectively.

Training of interventionists has been demonstrated to improve the consistency and effectiveness of the intervention, which leads to better patient engagement and adherence [37]. For example, a review that examined interventions addressing adherence to medication among adults with chronic conditions reported that training interventionists before they deliver interventions was associated with better outcomes in medication adherence [20]. The evidence provided in this review offers additional support for training interventionists. Pharmacists were the most common interventionists in the successful interventions identified. Pharmacists are experts in pharmacotherapy and patient education, and therefore best placed to deliver interventions aimed at promoting medication adherence [35].

All studies that explored patient outcomes in addition to medication adherence, such as blood pressure and blood glucose, reported significant changes in at least one of the variables assessed. This suggested that interventions targeting medication adherence could also lead to other improved health outcomes associated with improved medication‐taking. Lehmann et al. [38] emphasized the strength of the association between medication adherence and clinical outcomes. Several studies have associated improved clinical outcomes with improved medication adherence [3, 39, 40]. However, in this review, this association was inconsistent. The inconsistency could be attributed to other factors, such as lifestyle. Given that both these conditions are lifestyle‐related, these inconsistencies call for a more precise method of how medication adherence together with other factors such as lifestyle modification impact blood pressure and glucose control.

Despite the positive impact of the interventions, it is possible that there were factors in the studies that, if considered, may have further improved medication adherence. Duration since diagnosis is one such factor. Ofori‐Asenso et al. [41] examined statin adherence in patients aged ≥ 65 years and reported that the risk of non‐adherence was highest during the first year of starting treatment [41]. Hence, when evaluating the impact of interventions on medication adherence, it is important to take into account when the intervention is being delivered in the patient's medication‐taking journey.

It was noticeable that only Soto et al. [8] included social support and none of the studies addressed cost‐related issues. Social support is a significant influencer of medication adherence as it promotes psychological comfort and influences materialistic support [35, 42]. Cost‐related non‐adherence is a consistently reported challenge, particularly in developing countries [42, 43, 44]. Decreasing out‐of‐pocket costs through strategies such as decreasing co‐payments and referring patients to state‐ or company‐based assistance plans has the potential to combat cost‐related non‐adherence [12, 43, 44].

This review identified several limitations within the included studies, which should be taken into account when interpreting the findings. Although blinding participants and interventionists in face‐to‐face delivered intervention studies is impossible, blinding of outcome assessments can enhance the quality of the studies. However, the majority of articles in this review lacked sufficient information on blinding of outcome assessment. The combining of adherence without specifying medication adherence with regard to hypertension or diabetes obscures the impact of the interventions on individual conditions. Another key limitation of most studies included was the reliance on self‐reporting. Although self‐reported data can provide valuable insights into patients' experiences and behaviours, there are potential concerns regarding the accuracy and reliability of these data.

Interestingly, all studies [8, 12] that used objective methods of measuring adherence in this review did not report a significant improvement in medication adherence. However, De Leon et al. commented on the fact that they did not know whether the control group received any health information technology support and Soto et al. did not have a control group that served as a benchmark to measure the effect of an intervention. Due to the limitations of the study designs and available data, it is challenging to draw a definitive connection between objective methods of measuring adherence and the adherence results observed.

Future research could benefit from addressing these limitations by using blind data analysts, both objective and subjective measures of adherence and being disease‐specific when addressing/reporting adherence in patients with both hypertension and diabetes. Additionally, interventions should aim to target all five WHO determinants of non‐adherence [25], as adherence determinants are likely to interact and potentiate each other's influence.

Given that language and culture can influence effective strategies that improve medication adherence, such as patient education and communication [45], future studies should reinforce these by addressing language and cultural barriers in intervention studies. This will help create interventions that are more inclusive and effective for diverse patient populations.

The diversity of the strategies identified in the interventions reviewed aligns with the evidence from the literature, which suggests that a multifaceted approach is necessary to address medication adherence [27, 28]. Telephone and social media follow‐ups have become increasingly popular cost‐effective methods to maintain patient contact and provide ongoing support. For instance, a review by Cahaya and Skarayadi [46] showed a positive impact of eHealth on medication adherence in five studies and an insignificant impact in four studies. Thus, the effectiveness of strategies may vary depending on the context in which they are applied, underscoring the need for further research to identify the most useful strategies and corresponding settings.

To enhance the effectiveness of future studies targeting medication adherence in patients with both hypertension and diabetes, it is worth noting that most successful interventions in this review required continuous monitoring and engagement of pharmacists, which is not practical in routine clinical practice due to limited consultation time and a low clinician‐to‐patient ratio. To bridge this gap, other strategies can be considered, such as involvement of pharmacy technicians, community health workers (CHWs) or digital health innovations such as artificial intelligence (AI). Enhancing medication adherence through interprofessional collaboration between pharmacists and CHWs is proving to be a viable strategy. For instance, a study by Deidun et al. [47] reports on the use of Aboriginal CHWs to bridge the cultural gap between patients and pharmacists in a home medication review programme. Similarly, Wheat et al. [48] report on the development of a patient plan to address barriers to medication adherence following a CHW and pharmacist collaboration.

AI can be more widely incorporated into both monitoring and increasing medication adherence. For instance, a study that used AI to measure medication adherence, and provided medication reminders and dosing instructions, reported 100% adherence in the intervention group and 50% adherence in the control group [49].

Moreover, digital health tracking integrated with electronic health records can provide real‐time data on medication adherence and send automated alerts to healthcare professionals when a certain level of medication non‐adherence is detected. Although this proactive approach is promising, more research is needed to assess the long‐term effectiveness of digital health‐driven interventions in patients with coexisting hypertension and diabetes.

## 5 Conclusion

The use of multifaceted interventions involving pharmacists and fostering continuous interaction between healthcare providers and patients has the potential to improve medication adherence in patients with both hypertension and diabetes. However, research on interventions targeting medication adherence in patients with both hypertension and diabetes is still sparse, making definitive conclusions challenging.

Multifaceted interventions effectively address the factors influencing medication adherence by incorporating both informational and sociobehavioural determinants of medication adherence. It is evident from the review that pharmacists have the potential to be good intervention initiators due to their opportunities to directly interact with patients and their expertise in pharmacotherapy and patient education. Continuous engagement between healthcare providers and patients allowed for proactive identification and resolution of barriers to medication adherence, leading to better treatment outcomes.

## Author Contributions

**Pauline Tendai Maniki:** conceptualization, investigation, writing–original draft, methodology, validation, writing–review and editing, formal analysis, project administration, data curation. **Betty Bouad Chaar:** conceptualization, writing–review and editing, validation, methodology, formal analysis, supervision. **Parisa Aslani:** conceptualization, investigation, methodology, validation, writing–review and editing, formal analysis, supervision.

## Conflicts of Interest

The authors declare no conflicts of interest.

## Supporting information

Supporting information.

Supporting information.

## Data Availability

Data sharing is not applicable to this article as no new data were created or analysed in this study.
