# Supplementary material for: Impact of Interventions on Medication Adherence in Patients With Coexisting Diabetes and Hypertension
Source: Health Expect. 2024 Sep 9;27(5):e70010. doi: 10.1111/hex.70010 (PMC11381960; doi:10.1111/hex.70010)
Supplement: Supplementary file 1 — Supporting information. [file HEX-27-e70010-s001.docx]

| **Study** | **Method used to assess medication adherence** |
| --- | --- |
| **De Leon *et al.,* 2015^12^** | - Indirect – objective - The medication possession ratio (MPR) was used to measure medication adherence. - The MPR was determined by adding the total number of days of prescription supply dispensed between the first and last pharmacy fill, excluding the last fill, divided by the number of days between the prescriptions. - The yearly MPR was calculated and averaged across each therapeutic drug class. - Members were considered adherent if the average MPR was >80% |
| **Soto *et al.,* 2015^8^** | - Indirect – objective - Pill count - Patients received medication exceeding the quantity required before their next visit. Medication adherence was measured by calculating the percentage of pills remaining in the bottles issued to the patients.     Pill count = [(no. of pills dispensed - no. of pills returned) ÷number of pills prescribed] X 100.   - A pill count between 80 and110 % was considered good adherence |
| **Moorhead *et al.,* 2017^15^** | Direct   - Patients received sensor-enabled medicines to directly measure medication adherence and a wearable sensor to record medication activity. - They also received a digital health application for reminders and to confirm medication taking. - There was a web portal for the health care team to access patient data. - Patients entered their medication schedule i.e. medicine, dose, and frequency of administration into the digital health application. - Patients received push notifications at the time their medication was scheduled (dose time and not dosage information). - Medication adherence was measured using a binary variable that indicated if the patient took any medication after seeing or before seeing the reminder. - Overall adherence was calculated from the mean daily adherence which was defined as the number of pills   detected by the sensor patch divided by the number of pills expected for each medicine.   - Daily on-time adherence was also measured by dividing the number of pills detected by the sensor patch within two hours before or after the scheduled dose time by the number of pills expected for each medicine. |
| **Kwayke *et al.,* 2021^3^** | - Indirect-subjective - The MARS-10 questionnaire was used to categorize the level of medication adherence into adherent and non-adherent. |
| **Contreras-Vergara *et al.,* 2022^11^** | - Indirect-subjective - Medication adherence was evaluated using the Morisky medication adherence scale. - The total score was calculated by adding 8 scores together. - A score of 8 indicated high adherence, 6 to 7 – medium adherence, and <6 was considered low adherence. |
| **Malik *et al.,* 2022^2^** | - Indirect -subjective - Medication adherence was determined using the brief medication questionnaire. - The questionnaire consisted of 11 questions, divided into 3 screens namely; regimen screen, belief screen and recall screen. - The regimen screen asks patients about their medication administration over the past week. The belief screen addresses questions related to the medicine’s effects and side effects, while the recall screen includes questions related to remembrance of potential difficulties during medication administration. - A score of 1 is given if a patient reports being adherent and a score of 0 is given if a patient is not adherent. - A final score of 0 signifies no self-reported non-adherence or barriers to adherence and 4 indicates the presence of self-reported non-adherence. - A score ≥1 indicates a positive screen for a specific barrier. |
| **Wang *et al.,* 2022^6^** | - Indirect – subjective - Medication adherence level was determined using Morisky-Green test which consists of 4 questions. - Responding no was scored 1 point per question while yes was scored 0. - A score of 4 indicated adherence while 3,2 and 1 indicated non-adherence. |
