# Supplementary material for: Impact of Interventions on Medication Adherence in Patients With Coexisting Diabetes and Hypertension
Source: Health Expect. 2024 Sep 9;27(5):e70010. doi: 10.1111/hex.70010 (PMC11381960; doi:10.1111/hex.70010)
Supplement: Supplementary file 2 — Supporting information. [file HEX-27-e70010-s002.docx]

**Search strategy**

1. ('medication adherence' OR 'patient adherence' OR 'therapy adherence' OR

'treatment adherence' OR 'medication intake adherence' OR 'medication compliance' OR

'patient compliance' OR 'persistence' OR 'concordance' OR (Medication* adj3 (adher* OR comply OR compliance OR persist* OR non-adhere* OR nonadhere* OR noncompliance OR non-compliance OR nonpersistent OR non-persistant))) **AND**

1. ('interventions' OR 'strategies' OR 'programs') **AND**
2. ('diabetes' OR 'diabetes mellitus') **AND**
3. ('hypertension' OR 'high blood pressure')
4. Limit to articles published from June 2012 to December 2023
5. Limit to articles published in English
